# Taxonomic overview of *Polymixis serpentina* (Treitschke, 1825) species-group, with the description of a new species (Lepidoptera, Noctuidae, Xyleninae)

**DOI:** 10.3897/zookeys.201.3035

**Published:** 2012-06-14

**Authors:** Oleg Pekarsky

**Affiliations:** 1 H-1068 Budapest, Felsőerdősor u. 16-18, Hungary

**Keywords:** Lepidoptera, Noctuidae, *Polymixis serpentina* species-group, new species, Lebanon

## Abstract

The taxa of the *Polymixis serpentina* (Treitschke, 1825) species-group are revised. The external and genital features of all known taxa and a new species, *Polymixis ivanchiki* **sp. n.** (Lebanon, Israel, Turkey and Iran) are described and illustrated. *Polymixis serpentina iatnana* Hacker, 1996, is treated here as a species distinct from *Polymixis serpentina* (**stat. n.**). A diagnostic comparison of the members of the species-group is provided; descriptions of the genitalia of *Polymixis serpentina minoica* Fibiger, 1992 and *Polymixis iatnana* are given for the first time.

## Introduction

*Polymixis* Hübner, [1820] is a Palearctic genus of the subfamily Xyleninae. During the last thirty years several remarkable works have been published dealing with the taxonomic aspects of the genus (Beck 1996, 1999; Ronkay et al. 2001). In the latest revisionary work (Witt and Ronkay 2011) *Polymixis serpentina* is attributed to the subgenus *Polymixis* Hübner, [1820]. Actually, two insular subspecies are distinguished: *Polymixis serpentina minoica* from Crete and *Polymixis serpentina iatnana* from Cyprus; both were described based on external features only. The authors (Fibiger 1992; Hacker 1996) stated in the descriptions that the genitalia show no difference between the subspecies. The existing differences in genitalia structures of the three subspecies of *Polymixis serpentina* are described and discussed in detail in the present paper.

## Systematic part

### Polymixis ivanchiki sp. n.

urn:lsid:zoobank.org:act:37121E63-1CB5-49F7-97BF-1B256CD340DB

http://species-id.net/wiki/Polymixis_ivanchiki

Figs 1–2

#### Holotype.

Male, Lebanon N., Fnaideg env., h-1440m, 12.x.2007, leg. Krueger, Saldaitis; slide No.: OP0941m (coll. O. Pekarsky, deposited in the HNHM Budapest).

#### Paratypes.

Lebanon. 4 ♂♂, 1 ♀ Caza Dchare, Berhalioun, h-700m, 22.x.2008, leg. Punta, slide No: OP1352m (male) (coll. O. Pekarsky); 20 ♂♂, 6 ♀♀ from the same locality (coll. A. Floriani); 4 ♂♂, 1 ♀ Caza Dchare, Berhalioun, Dayrouna mt., h-1200 m, 24–25.x.2008, leg. E. Punta, (coll. A. Floriani); 1 ♀ Caza Aaakkar, Bezbina, h-700m, 26.x.2008, leg. Punta, (coll. O. Pekarsky); 1 ♂ from the same locality, (coll. A. Floriani); 2 ♀♀ Cedrus Mts., Aayoun Urghouch, h-2130m, 10.x.2007, leg. Krueger, Saldaitis; 1 ♀ Laqlouq env., h-1600m, 14.x.2007, leg. Krueger, Saldaitis, slide No: OP0942f (female) (coll. O. Pekarsky); 1 ♀ 60 km S v. Beirut, 30.X.1963, leg. E. & A. Vartian, slide No: OP1347f (female) (coll. NHMW). Turkey. 1 ♂, 2 ♀♀ Turkey, Prov. Hatay, 6 km N of Yayladagi, 1100 m, 36 00 E, 36 05 N, 20.x.1993, leg. Gy. Fábián, B. Herczig, Gy. László, K. Szeőke, slide No: OP1029f (female) (coll. G. Ronkay); 1 ♂, Prov. Urfa, Halfeti, valley of Euphrat, 500 m, 37°52'E, 37°14'N, 19.x.1993, leg. Gy. Fábián, B. Herczig, Gy. László, K. Szeőke, slide No: OP1428m (male) (coll. G. Ronkay). Israel. 1 ♂, N. Galilea, Nimrod, 800 m., 10–20.10.2004, ligh trap (coll. P. Gyulai), 2 ♂♂, Jerusalem, X.2006, leg. V. Kravchenko, slide No: OP1632m (male) (coll. O. Pekarsky), 4 ♂♂, 6 ♀♀ SW-Iran, Berge O v. Kasri Shirin, 24.x.1963, leg. Kasy & Vartian, slide Nos: OP1348m (male), OP1349f (female) (coll. NHM Vienna).

#### Diagnosis.

*Polymixis ivanchiki* is a sister taxon of *Polymixis serpentina* and is hardly distinguished externally from it, although the genitalia show clearly recognisable differences in both sexes. The forewing pattern of the two species is very similar, only the shape of reniform stigma show certain specific features. The reniform stigma of *Polymixis ivanchiki* is slenderer, more S-shaped with a finely lunulate inner dark line, whereas that of *Polymixis serpentina serpentina* is larger and more or less elliptical, with more parallel sides. The male genitalia of the two species are similar in most characters. The most significant difference between the two species is the shape of juxta. *Polymixis ivanchiki* has a characteristic anchor-like juxta, wide medially, and tapered evenly posteriorly into a long acutely angled process with a posteriorly tapering lateral process on each side with serrated inner edges at their tips; the juxta of *Polymixis serpentina* has wide posterior extension, less broad medial part and the two lateral arms lack the apical serration.

The most conspicuous difference between the female genitalia of two species can be found in the shape of the antrum. The posterior (anal) margin of the antrum of *Polymixis ivanchiki* has a very deep cleft, which reaches the middle of antrum, whereas *Polymixis serpentina* has only a slight, shallow cleft.

#### Description.

**Figures 1–8.**
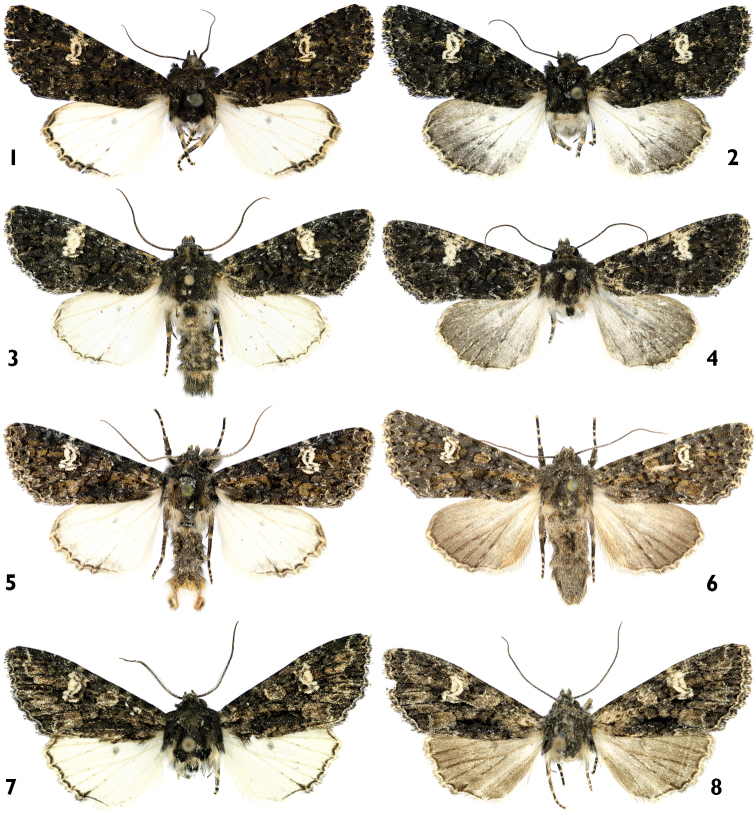
Adults. **1, 2** *Polymixis ivanchiki* sp. n. **1** holotype male, Lebanon **2** paratype female, Lebanon **3, 4** *Polymixis serpentina serpentina* **3** male, Slovenia **4** female, Slovenia **5, 6** *Polymixis serpentina minoica* **5** male, Greece, Crete **6** female, Greece, Crete **7, 8** *Polymixis iatnana* **7** paratype male, Cyprus **8**female, Cyprus.

Male (Fig. 1). Wingspan 38–40 mm, length of forewing 19 mm. Head, thorax and forewing dark brownish grey mixed with black hair-like scales. Forewing elongate, narrow; costa straight with white patches; outer margin with rather straight termen; most elements of forewing pattern rather blurred, except prominent white-defined reniform stigma; all but one crosslines black; basal line straight; subbasal line zigzagged; antemedial line waved; medial fascia a row of black streaks; postmedial line curved and dentate; subterminal line discontinuous, pale whitish, strongly sinuous, defined by short, black arrowhead spots at inner side; terminal line a row of tiny blackish triangles; orbicular stigma more or less rounded, formed by two black lateral arches filled with ground colour; reniform stigma large, white with narrow, black, lunulate inner line; fringes (cilia) as ground colour, finely chequered by whitish streaks at veins. Hindwing shining white with some black scales on veins, discal spot pale gray, terminal line black; cilia pale yellow mixed sparsely with black hair-like scales.

Female (Fig. 2). As male but with considerably darker suffused hindwing.

**Figures 9–14.**
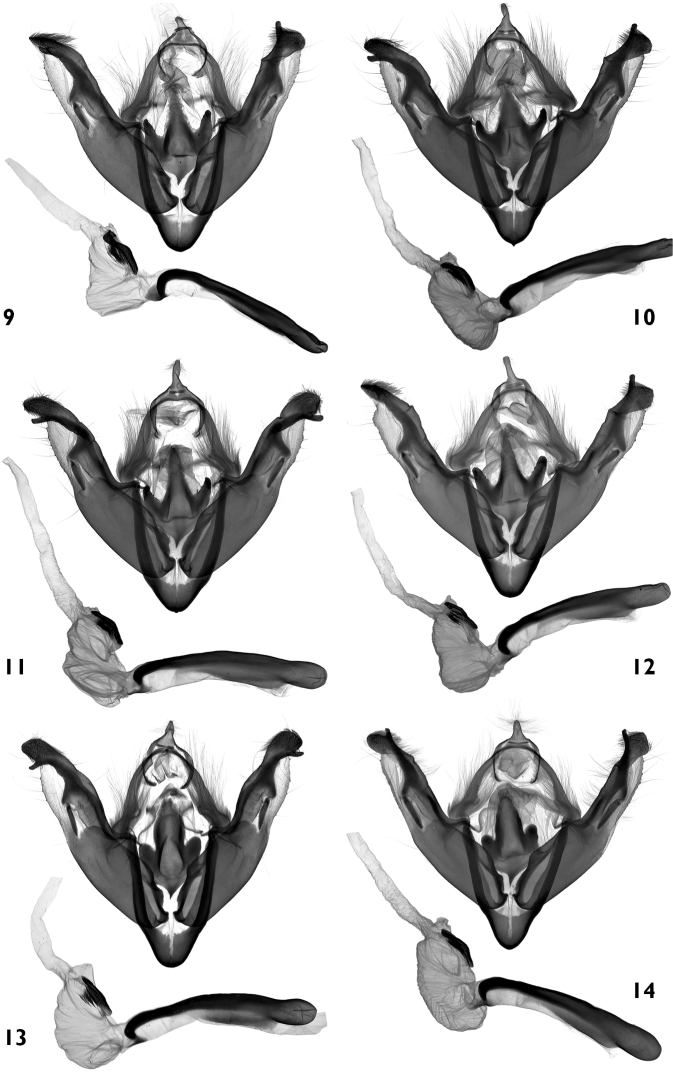
Male genitalia. **9–12** *Polymixis ivanchiki* sp. n. **9** holotype, Lebanon, slide No. OP0941m **10** paratype, Lebanon, slide No. OP1352m **11** paratype, SE Turkey, slide No. OP1428m **12** paratype, SW Iran, slide No. OP1348m **13, 14** *Polymixis serpentina serpentina* **13** Slovenia, slide No. OP0939m **14**  Croatia, slide No. OP1333m.<br/>

**Male genitalia** (Figs 9–12). Genital armature well-sclerotized; uncus weak, small and short, flattened and broad at base, tapering towards finely rounded and slightly hairy apex; tegumen broad, 0.67 times length of vinculum; penicular lobes small, rounded, bearing long setae; juxta anchor-like wide medially with two narrow and posteriorly tapered lateral arms with serrated inner edges towards apex; central posterior process long, thin, evenly tapering posteriorly; vinculum U-shaped. Valvae elongate with narrower distal half; cuculli asymmetrical, left one small and narrow, right one larger, apically more rounded, ventral surfaces densely setose on both cuculli; corona absent; sacculus large, elongate; clavus represented by a long, flat hump; clasper (harpe) straight, bar shaped; ampulla long, stick-like, with evenly tapered distal half and broad, reversed Y-shaped base. Aedeagus cylindrical, curved ventrally in distal part. Vesica tubular, everted forward then bent dorsad; basal tube short, subbasal bulb much wider, inflated, armed by narrow, elongated terminal field of spiniform cornuti; distal diverticulum small.

**Female genitalia** (Figs 25–27). Ovipositor medium-long, papillae anales elongated, hairy, with long setae on sides and shorter setae apically. Apophyses anteriores strong, with spatulate tips; apophyses posteriores thin and slightly longer than apophyses anteriores. Antrum (ostium bursae) large, broad, trapezoidal, strongly sclerotized, incised caudally forming deep cleft on ventral surface extending from posterior margin almost to middle of antrum. Ductus bursae flattened, semi-tubular, strongly sclerotized, medially folded and twisted. Appendix bursae conical, with sclerotized ribs and wrinkles; corpus bursae membranous, ovoid with four signum-stripes of different lengths.

#### Etymology.

The new species named in honour of Ukrainian zoologists, Ivanchik Taisiya Semenivna (1937-2007) and Ivanchik Grigoriy Semenovich (1929–2011), teachers of Department of Zoology, Faculty of Biology, Yuriy Fedkovych Chernivtsi National University.

#### Distribution.

The species is distributed in the Near East (Israel, Lebanon, SW Iran) and the southern parts of Turkey (provinces Hatay and Urfa).

### Polymixis serpentina serpentina

(Treitschke, 1825)

http://species-id.net/wiki/Polymixis_serpentina_serpentina

Figs 3, 4

#### Material examined.

Slovenia, slide Nos: ♂ OP0939m, ♀ OP0940f; Croatia, slide Nos: ♂ OP1333m, ♀♀ OP1334f, RL7061f; Macedonia, slide Nos: ♂ OP1445m, ♀ OP1446f; Bulgaria, slide Nos: ♂OP1455m, ♀ OP1335f; Greece, slide Nos: ♂♂ OP1014m, OP1336m, OP1338m, ♀♀ OP1015f, OP1337f, OP1339f; Italy, Puglia, slide Nos: ♂♂ OP1443m, OP1452m ♀♀ OP1444f, OP1453f; Greece, Rodos, slide No: ♂ OP1038m, ♀ OP1039f; Turkey, Nos: ♂♂ OP1035m, OP1037m, ♀♀ OP1028f, OP1036f.

#### Male genitalia

**Figures 15–20.**
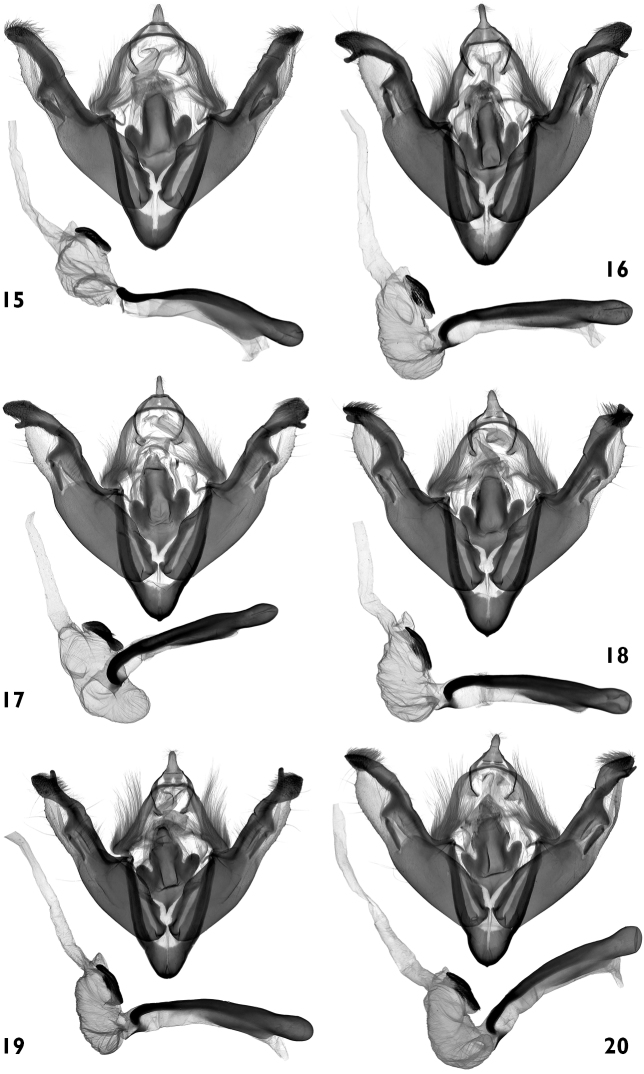
Male genitalia. **15–20** *Polymixis serpentina serpentina* **15** Central Greece, slide No. OP1014m **16** Greece, Rhodos, slide No. OP1038m **17** Turkey, Antalya prov., slide No. OP1035m **18 **Turkey, Ankara prov., slide No. OP1037m **19** Italy, Puglia, slide No. OP1443m **20** Bulgaria, slide No. OP1455m.

(Figs 13–20)**.** A detailed description of genitalia of this taxon is given by Ronkay et al. (2001). To this text it is possible to add only some more details of the characteristic structure of the juxta. In the typical populations of *Polymixis serpentina serpentina* from Croatia the juxta has a wide posterior extension and medial part has a posterolaterally directed lobe or lateral arm on each side without any serration.

#### Female genitalia

(Figs 28–36)**.** Described by Ronkay et al. (2001). In addition that description, it is worth highlighting the importance of the shape of the posterior margin of the antrum, which has only a slight, rounded concavity in the middle. This shape of antrum is characteristic for the *Polymixis serpentina serpentina* populations from Croatia (type locality) and adjacent areas (Slovenia, Bulgaria) (Figs 28–30). Females from the more eastern areas (north and central Greece, central and south Turkey) have the posterior margin of antrum more deeply incised (Figs 31–36). It should be noted that the genitalia figure in *Noctuidae Europaeae* 5 (fig. 141) shows the male genitalia of *Polymixis serpentina minoica*, whereas fig. 335 illustrates the female genitalia of the nominate subspecies.

#### Note.

Moths from Rhodes Island have some slight differences in male genital structure; further studies on a larger sample of material are needed to clarify the taxonomic situation of this insular population. It is not impossible that this population represents another, as yet undescribed, subspecies of *Polymixis serpentina*.

**Figures 21–24.**
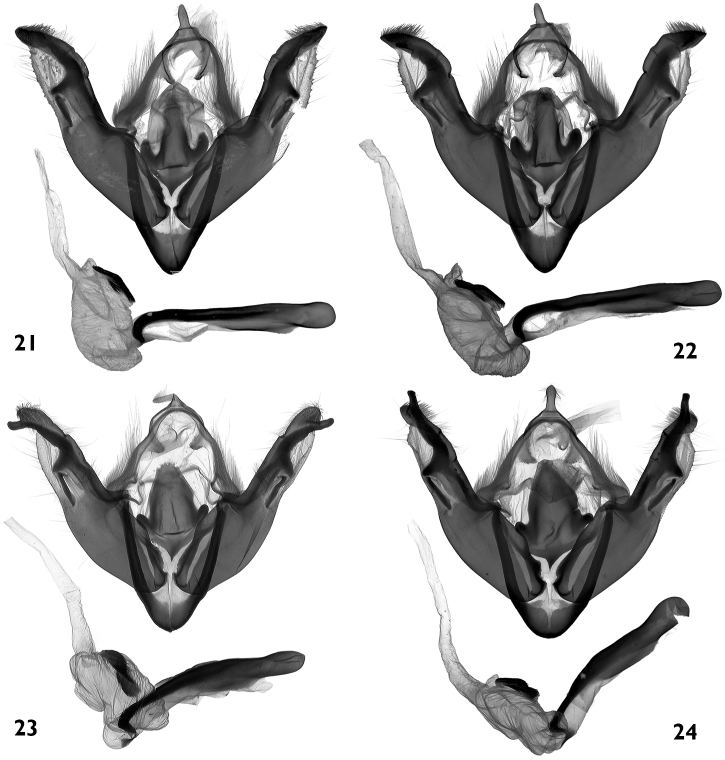
Male genitalia. **21, 22** *Polymixis serpentina minoica* **21** Greece, Crete, slide No. OP1283m **22** Greece, Crete, slide No. OP1345m **23, 24** *Polymixis iatnana* **23**Cyprus, slide No. OP1030m **24** Cyprus, slide No. OP1282m.

### Polymixis serpentina minoica

Fibiger, 1992

http://species-id.net/wiki/Polymixis_serpentina_minoica

Figs 5, 6

#### Material examined.

Greece, Crete slide Nos: ♂♂ OP1283m, OP1345m, ♀♀ OP1284f, OP1340f, OP1346f.

#### Male genitalia

(Figs 21, 22)**.** The genitalia of this taxon were not described in the original description. It was stated that there were no differences between the two subspecies. There are, however, some recognisable differences between the structure of the genitalia of the Cretan and the other populations of *Polymixis serpentina*. The ground plan of the male genitalia of *Polymixis serpentina minoica* and the nominotypical subspecies is the same, but the shape of the juxta is different and all parts of the posterior half of valvae are more massive, being more intensively sclerotized. The posterior extension of juxta is similar to that of *Polymixis serpentina serpentina*, but its lateral arms are more elongated, slightly curved, and significantly more divergent from the basal plate. The valvae are wider, and the costa, triangular basal plate, and its extension, appear rough and massive due to their stronger sclerotization; *Polymixis serpentina serpentina* has narrower valvae with all parts of their distal sections being finer and thinner.

#### Female genitalia

(Figs 37–39)**.** The copulatory organ of ssp. *minoica* differ from those of the ssp. *serpentina* in the on average shallower concavity on the posterior margin of antrum.

**Figures 25–30.**
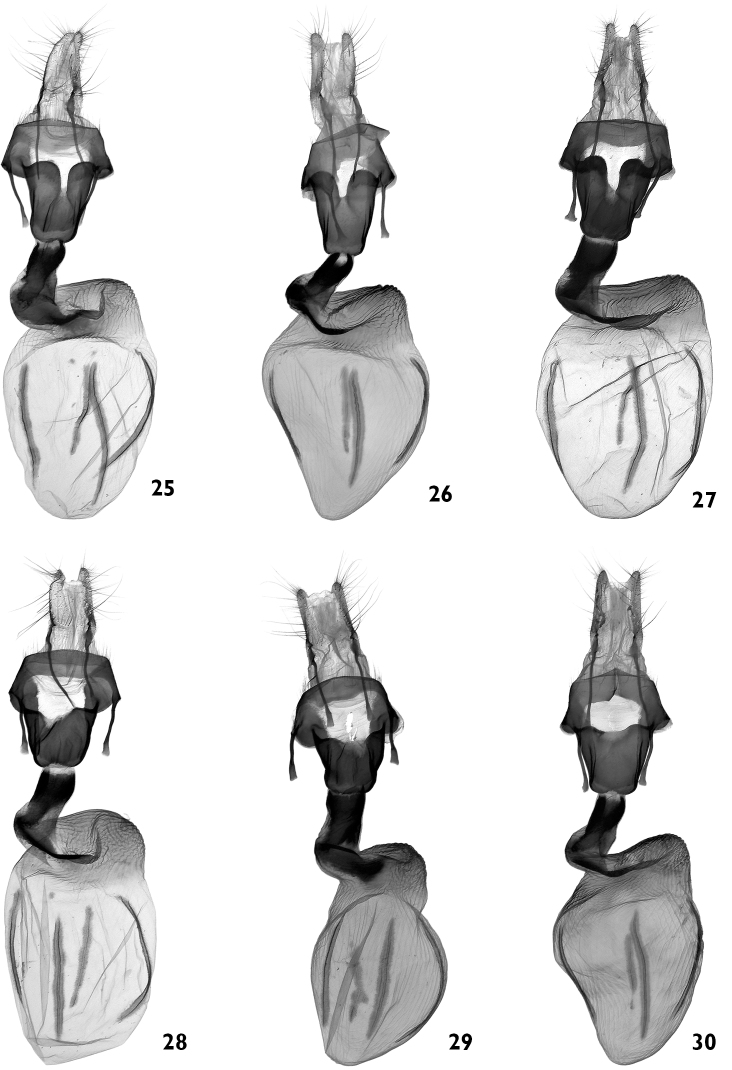
Female genitalia. **25–27** *Polymixis ivanchiki* sp. n. **25** paratype, Lebanon, slide No. OP0942f **26** paratype, Lebanon, slide No. OP1347f **27** paratype, SE Turkey, slide No. OP1029f **28–30** *Polymixis serpentina serpentina* **28** Slovenia, slide No. OP0940f **29** Croatia, slide No. OP1334f **30** Bulgaria, slide No. OP1335f.

**Figures 31–36.**
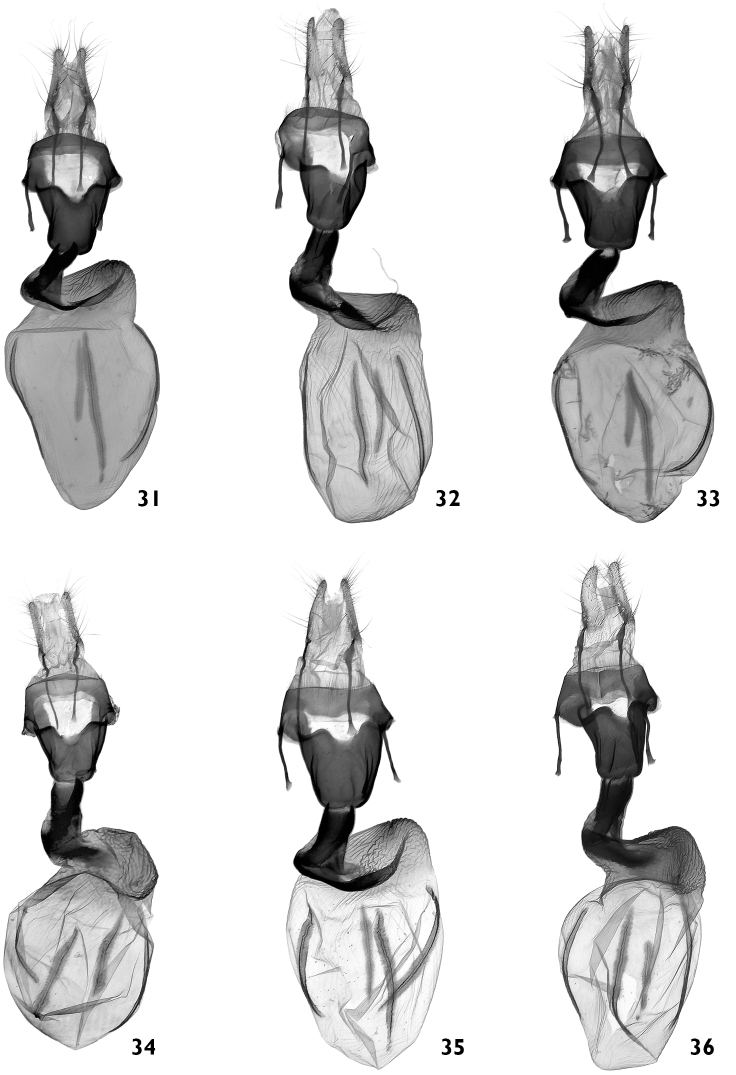
Female genitalia. **31–36** *Polymixis serpentina serpentina* **31** NE Greece, Kirki, slide No. OP1339f **32** Central Greece, Amfissa, slide No. OP1015f **33** Greece, Peloponnes, slide No. OP1337f **34** Greece, Rhodos, slide No. OP1039f **35** Turkey, Antalya county, slide No. OP1036f **36** Turkey, Icel county, slide No. OP1028f.

### Polymixis iatnana

Hacker, 1996 stat. n.

http://species-id.net/wiki/Polymixis_iatnana

Figs 7, 8

#### Material examined.

Cyprus, Slide Nos; ♂♂ OP1030m Paratype, OP1282m, ♀♀ OP1031f, OP1041f.

#### Male genitalia

(Figs 23, 24)**.** The original description contains the following text about the genitalia structure: “Ohne Berücksichtigung der sowohl beim Männchen als auch beim Weibchen nahezu identischen Genitalstrukturen würde man sie für eine gut ausgeprägte Art betrachten“. The genitalia of both sexes show, however, clearly visible differences, especially in shape of juxta and antrum. *Polymixis iatnana* has a wide, shield-like juxta with a wide posterior extension and very small drop-like lateral arms.

#### Female genitalia

(Figs 40, 41)**.** The female genitalia are characterized by the very wide and shallow concavity on the posterior margin of the antrum, which extends from one lateral edge to the other, whereas this incision is in the middle of the posterior margin in the two other subspecies.

**Figures 37–41.**
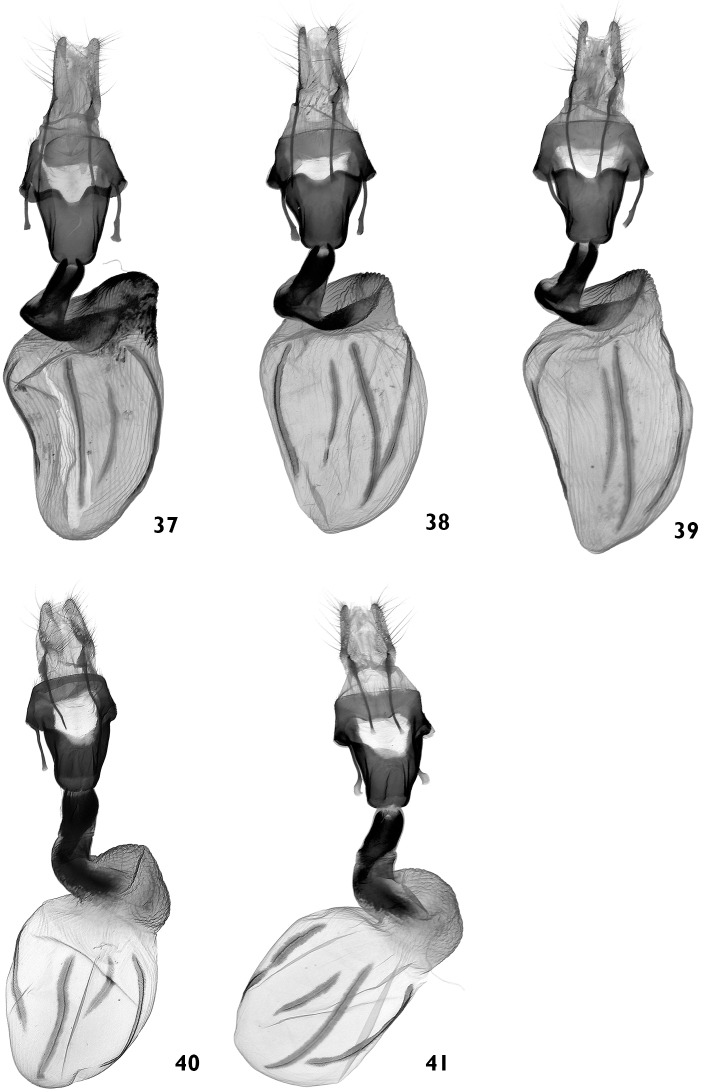
Female genitalia. **37–39** *Polymixis serpentina minoica* **37** Greece, Crete, slide No. OP1284f **38** Greece, Crete, slide No. OP1340f **39** Greece, Crete, slide No. OP1346f **40, 41** *Polymixis iatnana* **40** Cyprus, slide No. OP1031f **41** Cyprus, slide No. OP1041f.

## Supplementary Material

XML Treatment for Polymixis ivanchiki

XML Treatment for Polymixis serpentina serpentina

XML Treatment for Polymixis serpentina minoica

XML Treatment for Polymixis iatnana
